# Natural Enemies, Mediated by Landscape and Weather Conditions, Shape Response of the Sorghum Agroecosystem of North America to the Invasive Aphid *Melanaphis sorghi*

**DOI:** 10.3389/finsc.2022.830997

**Published:** 2022-04-12

**Authors:** Michael J. Brewer, Norman C. Elliott, Isaac L. Esquivel, Alana L. Jacobson, Ashleigh M. Faris, Adrianna Szczepaniec, Blake H. Elkins, J. W. Gordy, Adrian J. Pekarcik, Hsiao-Hsuan Wang, Tomasz E. Koralewski, Kristopher L. Giles, Casi N. Jessie, William E. Grant

**Affiliations:** ^1^Department of Entomology, Texas A&M AgriLife Research, Corpus Christi, TX, United States; ^2^Plant Science Research Laboratory, US Department of Agriculture-Agricultural Research Service, Stillwater, OK, United States; ^3^Department of Entomology and Plant Pathology, Auburn University, Auburn, AL, United States; ^4^Department of Entomology, Texas A&M University, College Station, TX, United States; ^5^Department of Agricultural Biology, Colorado State University, Fort Collins, CO, United States; ^6^Syngenta Crop Protection, Greensboro, NC, United States; ^7^Department of Ecology and Conservation Biology, Texas A&M University, College Station, TX, United States; ^8^Department of Entomology & Plant Pathology, Oklahoma State University, Stillwater, OK, United States

**Keywords:** *Melanaphis sacchari*, invasive species management, ecological modeling, ecosystem services, biological control, agroecosystem resilience

## Abstract

The sorghum (*Sorghum bicolor* [L.]) agroecosystem of North America provided an opportunity to evaluate agroecosystem response to an invading insect herbivore, *Melanaphis sorghi* (Theobald) (sorghum aphid) (previously published as *Melanaphis sacchari* Zehntner) (Hemiptera: Aphididae) onto a widely planted crop that experiences a range of agro-landscape and weather conditions. Initial sorghum risk assessments after *M. sorghi*'s invasion in the mid-2010s provided forecasts of range expansion and annual migration, which were based on aphid life history, extent of sorghum cultivation and susceptibility to *M. sorghi*, and weather (aphid-plant-weather [APW] risk scenario). A more comprehensive risk assessment proposed here brings top-down forces of *M. sorghi*-natural enemy interactions to the forefront as mediated by agro-landscape and weather conditions (aphid-enemy/landscape-weather mediated [AE/LW] risk scenario). A hypothesis of regional differences in aphids and natural enemies and sensitivity to agro-landscape and weather was tested using empirical data of insect, landscape, and weather data across 5 years and four regions (two in the U.S. Great Plains [South GP and North GP], one farther south (South), and one in the southeast U.S. [South E]). Natural enemies were widespread with two parasitoids and four coccinellid species common across regions, but regional variation in *M. sorghi* and natural enemy abundance was detected. The AE/LW risk scenario accounted for natural enemy abundance and activity that was highest in the South region, functioned well across agro-landscape and weather conditions, and was accompanied by average low *M. sorghi* abundance (~23 *M. sorghi* per leaf). Positive correlations of natural enemy-*M. sorghi* abundance also occurred in the South GP region where *M. sorghi* abundance was low (~20 *M. sorghi* per leaf), and selected natural enemy activity appeared to be mediated by landscape composition. *Melanaphis sorghi* abundance was highest in the South E region (~136 aphids/leaf) where natural enemy activity was low and influenced by weather. The AE/LW risk scenario appeared suited, and essential in the South region, in assessing risk on a regional scale, and sets the stage for further modeling to generate estimates of the degree of influence of natural enemies under varying agro-landscape and weather conditions considered in the AE/LW risk scenario. Broadly, these findings are relevant in understanding agroecosystem resilience and recommending supportive management inputs in response to insect invasions in context of natural enemy activity and varied environmental conditions.

## Introduction

The sorghum, *Sorghum bicolor* (L.), agroecosystem of North America provided an opportunity to evaluate agroecosystem response to an invading insect herbivore onto a widely planted crop that experiences a range of agro-landscape and weather conditions. Nearly all cultivation of sorghum in the United States of America (U.S.), Mexico, and other countries in the vicinity of the Gulf of Mexico became at risk to *Melanaphis sorghi* Theobold (sorghum aphid) (Hemiptera: Aphididae) infestations during the mid-2010s onward, as indicated by overlaying *M. sorghi* infestations with sorghum production (1, 2). A superclone designation of the genotype found on sorghum (3, 4) and the classification of this invasive genotype as *M. sorghi* with likely origin from Africa or Asia (5) was supported by morphometric and molecular research. Previous publications of *M. sorghi* on sorghum in North America have used the name *Melanaphis sacchari* Zehntner [(6), and references therein]. This aphid principally damages sorghum (7) and is suppressed by natural enemies in subtropical and tropical regions (8).

*Melanaphis sorghi* was not a significant sorghum pest in North America until outbreaks on sorghum began to be detected along the Texas Gulf Coast in 2013 (2). From 2013 to 2015 *M. sorghi* spread rapidly. The near-continental scale of the invasion extended south into Mexico and the Caribbean islands. Northward, its range extended to the central U.S. (Illinois and Kansas) and along the eastern coast from Florida to North Carolina. It also was detected to lesser extents in Arizona and California (2, 6, 9). The rapid spread and damaging outbreaks supported its characterization as a novel, invasive colonizer causing substantial ecological disruption and economic losses (10). Initial success of *M. sorghi* as an invader may be attributable to sorghum as a widely available resource and excellent *M. sorghi* host [i.e., resource availability hypothesis (11) and defense-free space hypothesis (12)], few or ineffective top-down forces imposed by pre-existing aphid natural enemies [i.e., enemy-free space hypothesis (13)], suitability to climate and other abiotic factors (13, 14), and the number of source aphids initially introduced and radiating outward after establishment in the Gulf Coast area [propagule pressure hypothesis (15)], or some combination of these factors (16, 17).

From the perspective of agroecosystem response and resiliency to disturbances caused by insect invasions, sorghums in cultivation were largely lacking in bottom-up contraints to *M. sorghi* as most were susceptible to *M. sorghi* with damage resulting in economic loss (2, 18) and supported high *M. sorghi* reproduction which provided source populations for range expansion (19, 20). Potential top-down forces in *M. sorghi* population regulation were also a point of discussion during the establishment and early expansion phase of the invasion. The periodic outbreaks of *M. sorghi* on sorghum overlapped in geographic range with a suite of natural enemies suppressing other aphids occurring in cereal grains grown in the North American Great Plains (6). *Melanaphis sorghi* on sorghum presented an abundant prey item to add to the aphid herbivore guild on sorghum: *Rhopalosiphum maidis* (Fitch) (corn leaf aphid), *Sipha flava* (Forbes) (yellow sugarcane aphid), and *Schizaphis graminum* (Rondani) (greenbug) (Hemiptera: Aphididae) (21). Within a few years of its introduction, natural enemies preying on *M. sorghi* consisted of about 19 species including solitary endoparasitoids (Hymenoptera: Aphelinidae, Braconidae), a hyperparasitoid (Hymenoptera: Encyrtidae), lady beetles (Coleoptera: Coccinellidae), hoverflies (Diptera: Syrphidae), lacewings (Neuroptera: Chrysopidae and Hemerobiidae), and pirate bugs (Hemiptera: Anthocoridae) [e.g., (22–26)]. Maxson et al. (26) confirmed development to adults of many of these species fed *M. sorghi* in the laboratory. Selected lady beetle and lacewing species have been shown to suppress *M. sorghi* in small experimental arenas (27). Yet these observations were occurring in the background of rapid range expansion of *M. sorghi* and reports of *M. sorghi* outbreaks from the North American Great Plains to the southeastern U.S. (2).

Looking back at past cereal aphid invasions in the North American Great Plains, the most recent mass field releases of exotic natural enemies consisted of multiple species of parasitoids and predators released for control of *Diuraphis noxia* (Mordvilko) (Hemiptera: Aphididae) (Russian wheat aphid) during the later 1980s and early 1990s. Outcomes of these releases suggested that the natural enemy complex preying on *D. noxia* may be flexible in responding to future aphid invasions onto cereal grains (28). This proposition was based on data indicating that several endemic and long-time resident natural enemies along with a few intentionally introduced species were suppressing *D. noxia* and *S. graminum* on wheat in at least part of their range (6, 29, 30). Further, cereal aphid-natural enemy interactions may be influenced by agro-landscape and weather conditions that vary across geographic gradients (30–33). For example, aphid-enemy interactions on cereal grains occur where crop production extends across cool temperate to subtropical climatic zones of North America (6). A latent period of natural enemy response to *M. sorghi* as a new prey item would help explain the apparent low top-down forces during the establishment and early expansion phases of the invasion.

Ecological modeling approaches have been employed for forecasting agroecosystem disruption by and response to invasive species after establishment (34). Value of such efforts includes predicting range expansion, agricultural impact and need for management interventions, and effects of biotic and abiotic factors (35). For the *M. sorghi* invasion of sorghum in North America, ecological modeling and other qualitative sorghum risk evaluations initially focused on aphid life history, extent of sorghum cultivation and susceptibility to *M. sorghi*, and weather (2, 19, 20), which is termed here as the aphid-plant-weather (APW) risk assessment scenario. Host plant considerations in this assessment have included sorghum as an excellent reproductive host of *M. sorghi* until the plant deteriorates (36) and *M. sorghi* occurring on sorghum regrowth in harvested grain fields, forage sorghum fields, and the alternative host *Sorghum halepense* (L.) Pers. (johnsongrass) (37). These plants persist through winter in subtropical areas, providing harborage to *M. sorghi* (37). These characteristics suggested the persistent high risk of *M. sorghi* outbreaks in subtropical growing areas of the most southern U.S. (inclusive of two regions of this study) and countries farther south. Models also suggested a northward progression of declining risk characterized by more episodic outbreaks as winter host plant harborage declined in temperate zones and annual *M. sorghi* migrations into regions with expansive plantings of sorghum appeared driven by capacity of the aphid to move long distances *via* seasonal wind-aided long-distance flights (19).

A more comprehensive aphid-enemy/landscape-weather mediated (AE/LW) risk assessment scenario brings top-down forces of *M. sorghi*-natural enemy interactions to the forefront as mediated by agro-landscape conditions (partly as an indicator of bottom-down forces) and weather conditions (as a measure of general abiotic influences). This risk scenario was based on reports of natural enemy activity associated with *M. sorghi* that appeared to vary across regions. Field populations of *Aphelinus* sp. (Hymenoptera: Aphelinidae), syrphid larvae, and coccinellids suppressed *M. sorghi* placed on potted sorghum in Kansas (22). In south and central Texas, *Aphelinus nigritus* Howard (Hymenoptera: Aphelinidae) became the numerically dominant natural enemy attacking *M. sorghi*, and suppression was indicated by repeated measurements of *M. sorghi* and *A. nigritus* across the growing season (26). *Lysiphlebus testaceipes* (Cresson) (Hymenoptera: Braconidae) parasitized *M. sorghi* in northeastern Mexico (25), was infrequently observed parasitizing *M. sorghi* in Texas and Georgia (26, 38), and was not observed parasitizing *M. sorghi* in Kansas (22). Coccinellids and selected other predator taxa were commonly reported from south Texas (26) and Mexico (39) but were observed sporadically in Georgia (38). Prior to the *M. sorghi* invasion, variability in abundance of the natural enemy complex and in corresponding cereal aphid suppression has been reported in wheat and barley across the Great Plains (29, 30).

To compare expectations of the APW and AE/LW risk scenarios with empirical data, we pursued a large-region comparative study to characterize the natural enemy complex, estimate *M. sorghi* and natural enemy population trends, and detect relationships of natural enemy activity on *M. sorghi* as mediated by agro-landscape and weather conditions. Four sorghum production regions of North America were selected that experienced *M. sorghi* outbreaks on sorghum to varying degrees (23, 38, 40) and represented a range of subtropical and temperate climatic zones, intensity of sorghum production, and landscape structure. The working null hypothesis was *M. sorghi* and natural enemy population trends did not differ among regions and were insensitive to agro-landscape and weather conditions. If differences across the four regions were detected, the results were used to consider which of the two risk scenarios were most representative of the *M. sorghi* invasion and better represented agroecosystem response to our model system of an invading insect herbivore into a widely planted crop.

## Materials and Methods

### Sampling Regions and Techniques

**Figure 1:**
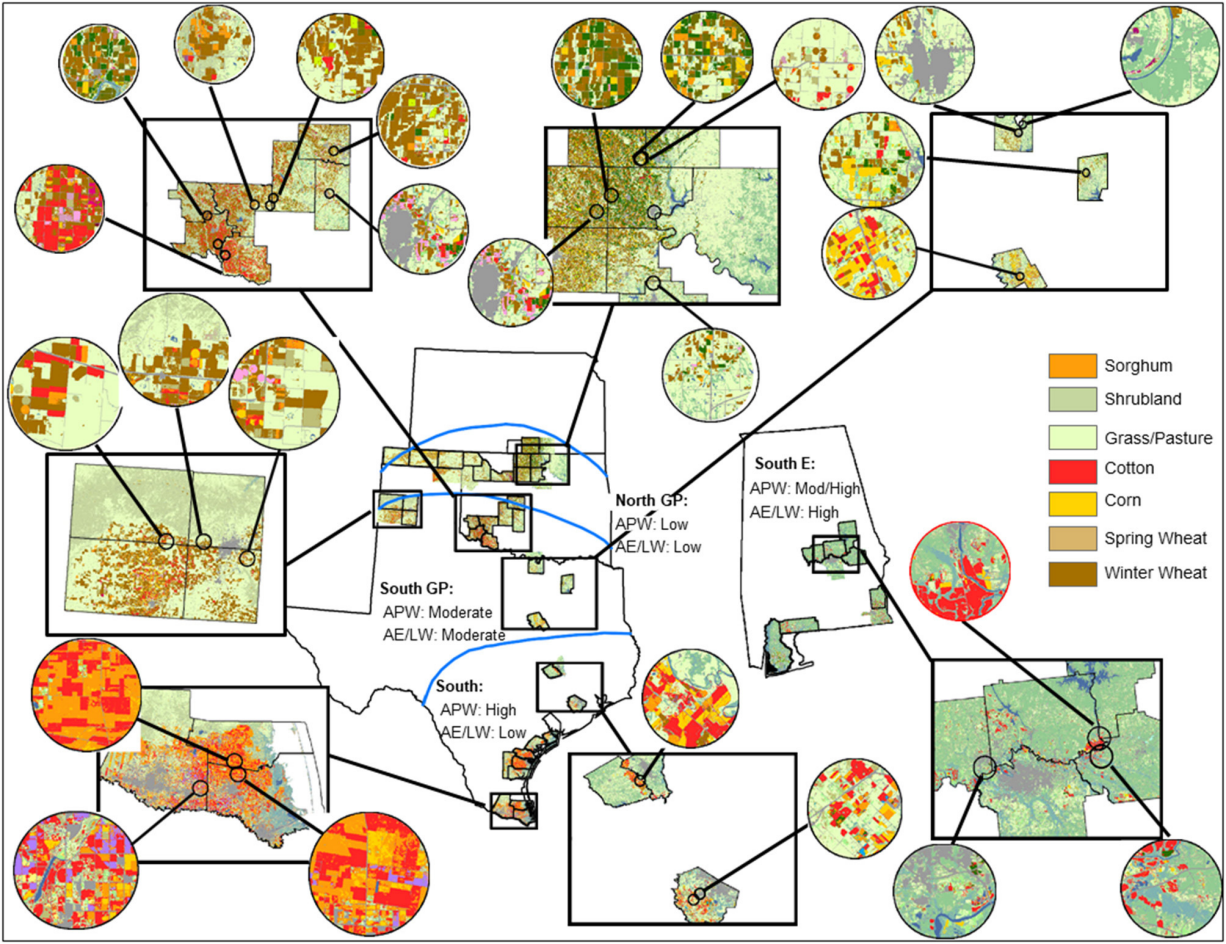
Areas sampled for *Melanaphis sorghi* and natural enemies across four regions (South, South GP, South E, North GP) generally following a latitudinal transition from subtropical, warm temperate, to temperate climates. Five kilometer radius buffers surrounding sampling locations (circle cut-outs) from counties visited (box cut-outs) are magnified to show the range of agro-landscape features encountered. Land cover symbology (see legend for color-coding) is taken from the website Cropscape for 2018 (41). Risk to *M. sorghi* (high to low) were assigned by expectations based on the aphid-plant-weather (APW) and aphid-enemy/landscape-weather mediated (AE/LW) risk scenarios (placed below the region labels). See text for rationale of risk assignments.

Natural enemy and aphid sampling occurred in sorghum fields cultivated for grain at multiple locations within four regions. The regions represented the extensive cereal grain production area of the U.S. portion of the North American Great Plains where *M. sorghi* quickly spread into widely planted sorghum (two regions labeled South GP and North GP) and its southern extension that included areas of first reported *M. sorghi* outbreaks where sorghum was a major agro-landscape component (one region labeled South). A southeast U.S. region was included where rapid eastward spread of *M. sorghi* occurred and sorghum was a lesser component of the agro-landscape (one region labeled South E) (Figure 1). The five-year study (2015–2019) overlapped with, but primarily followed, *M. sorghi*'s rapid geographic expansion from 2013 to 2015 (2). The specific geographies and climates were the Texas Gulf Coast extending from the Rio Grande Valley of southern Texas to near the city of Houston with primarily subtropical temperate climate (South), central Texas to the lower Texas Panhandle and across central Oklahoma with a warm temperate climate (South GP), south and central Alabama with a mix of subtropical and warm temperate climate (South E), and the upper Texas and Oklahoma Panhandles through northern Oklahoma and adjacent southern Kansas with a temperate climate (North GP) (Figure 1).

Sorghum fields sampled were in 40 counties within these four regions and were randomly selected from a list of probable *M. sorghi*-infested fields provided by local collaborators. Data were taken from multiple fields in each county. On average, per county and year, 3,073 leaves were inspected in 2.5 fields and eight sampling events (numbers of fields and sampling events are given in Table 1). Plant growth stage ranged from mid-vegetative growth through early grain fill stages of reproductive growth and was recorded as a whole field average (Supplementary Material 1). Sampling began when *M. sorghi* presence was confirmed. Average *M. sorghi* densities typically fell within the range of 20 and 70 *M. sorghi* per leaf, which bridged across unlikely to probable sorghum injury and risk to grain production [i.e., 40 *M. sorghi* per leaf is a commonly used indicator of expected economic injury (18)]. For each sampling event, *M. sorghi* and natural enemy taxa were counted on two leaves of each plant: the first green leaf toward the base of the plant and the uppermost unfurled leaf below the flag leaf (23). Plants were selected randomly, and no fewer than 60 leaves were inspected per sampling event. The length of inspection time was 20 min or more, with more time spent when aphid and natural enemies were common. For each field, first date of detection of *M. sorghi* was recorded and initiated two to five sampling events. Raw data were aggregated across sampling events and fields for each county and year, resulting in 87 data records used for analyses of this study (Table 1, Supplementary Material 1). Four data records of the South and South GP regions were derived from previous research (26).

**Table 1:** Sampling history in four regions where *Melanaphis sorghi* and its natural enemies were identified and enumerated from 2015 to 2019.

**Region^a^**	**Years**	**Yr-county^b^**	**Yr-county-field^b^**	**Yr-county-field-event^b^**	**Leaves inspected^b^**
South	4 (2016–19)	21	70	208	123,402
South GP	4 (2015–18)	27	51	190	64,088
South E	4 (2015–19)	9	11	42	26,430
North GP	3 (2017–19)	30	81	283	53,351

a *South, Texas coastal areas from Rio Grande Valley to near Houston; South GP, central Texas to lower Texas Panhandle and across central Oklahoma; North GP, upper Texas and Oklahoma Panhandles through northern Oklahoma and adjacent southern Kansas; South E, southern and central Alabama.*

b *At least 60 leaves were inspected during a sampling event. Data were aggregated for analyses by year-county combinations (n = 87 data reports) taken from multiple fields in a county*.

Infrequent detections or differences in the relative proportions of natural enemy counts across taxa did not appear to be affected by sampling method biases. There were no significant differences of the relative proportions of each natural enemy taxa recovered using the two-leaf inspection method adopted by this study to maximize efficiencies compared with a whole plant destructive sampling method. The results (contingency table analyses (42) in Supplementary Material 2) did not reveal differences in the sensitivity of the two methods in detecting parasitoids (mummies) and predators (adults and larvae), or in detecting differences across lower taxa used in this study (see next section). Also, the comparative study focused on regional comparisons of natural enemy abundance and activity and not cross-taxa comparisons, further reducing the concern of sampling method bias across natural enemy taxa. Therefore, the two-leaf sampling method was considered satisfactory for the large region comparative study.

### *Melanaphis sorghi* and Natural Enemy Taxa

Insects selected for analyses of the large-region comparison were *M. sorghi, A. nigritus* mummies, *L. testaceipes* mummies, lady beetle larvae, lady beetle adults, syrphid larvae, and lacewing (chrysopids and hemerobiids) larvae. *Melanaphis sorghi* was the dominant aphid species, with sporadic early season detections of *R. maidis, S. graminum*, and *S. flava*. The natural enemy taxa represented those previously identified from the U.S. and Mexico [e.g., (22–26)]. A species check list was generated for each region, with infrequently detected taxa limited to family-level classification. Periodically, starting in 2015, subsets of blue-black non-swollen aphid mummies were reared to adults and identified as *A. nigritus* based on comparison to voucher specimens [Texas A&M University Insect Collection, voucher #723 (26)]. Similarly, starting in 2017, subsets of light-brown swollen aphid mummies were reared to adults and identified as *L. testaceipes* based on taxonomic keys specific to parasitoids of cereal aphids (43). Using photographs and references in Maxson et al. (26), adult and larval lady beetles were identified to species during most sampling events, and syrphid and chrysopid larvae were identified to species in the South and South GP regions in 2015 and 2016.

Absolute abundance metrics for *M. sorghi* and natural enemy taxa were reported on a per leaf basis (density). A natural enemy activity metric was calculated as abundance per leaf divided by the number of live un-mummified aphids per leaf (and multiplied by 100 to aid presentation). For parasitoids, this activity metric provided a rough estimate of parasitism and was consistent with the predator activity metric. It can be recalculated to apparent field parasitism by including mummified aphids in the divisor, although this metric also underestimated parasitism because some un-mummified aphids were likely parasitized (44).

### Agro-Landscape and Weather Metrics

Agro-landscape composition metrics were selected to represent the crop directly disrupted by the invasive pest (sorghum), and cropland, grassland, and shrubland known to harbor natural enemies of cereal aphids (30–33). To calculate the metrics, georeferenced cropland data layers from 2015 to 2019 corresponding to the 87 year-county data records were managed within a geographic information system (GIS) using ArcMap 10.7 [ESRI, Redlands, CA (45)]. Cropland data layers were downloaded in raster format by state (i.e., Alabama, Kansas, Oklahoma, and Texas) from the website Cropscape (41) and imported into GIS. Circular buffers (5 km radius) were centered on the approximate centroid of each individual field sampled (Figure 1). The 5 km radius was selected to balance preferences for smaller scales appropriate to activity of parasitoids of cereal aphids [e.g., from 2 to 3 km radius (32, 33)] and larger scales more appropriate for activity of predators of cereal aphids (31). Using zonal statistics (45), total numbers of pixels for selected landscape metrics were calculated for each buffer. Overlap of buffers was infrequent and minimal (Figure 1). The agro-landscape composition metrics considered were percent sorghum (percent of pixels classified as sorghum) and percent cropland (percent of pixels associated with corn, cotton, sorghum, other small grains, legumes, and non-alfalfa hays). Two other metrics consisted of lesser managed lands: percent grassland-pasture (percent of pixels associated with various grass species and herbaceous vegetation managed for grazing and hay crops) and percent shrubland (percent of pixels associated with woody shrubs <5 m in height including young or stressed trees and interspersed with grasses) (46).

Weather metrics were calculated using data archived from the nearest weather station to each field and corresponding to each sampling event from 2015 to 2019 (47). Weather stations accessed were commonly at county airports, recording above ground (3 to 10 m) temperature. Data for analyses were calculated as averages from the first to last sampling event: minimum temperature (°C) averaged across daily lowest temperatures, maximum temperature averaged across from daily highest temperatures, and rainfall (cm) summed across daily amount of rainfall. The agro-landscape and weather metrics were merge-matched with the 87 year-county data records for further analysis (Supplementary Material 1).

### Data Analysis

Analysis of variance (Proc GLM, 48) was used to detect differences in the insect abundance and activity metrics across the four regions. Year was set as a blocking term to aid in partitioning yearly variation associated with weather and insect abundance that may affect ability to detect regional differences. Replication (i.e., data records) ranged from 21 to 30 for three regions (Table 1). Fewer year-county records were available in the South E region (*n* = 9), but the region was retained given its contrast with the other regions and the comparable sampling effort that occurred within yr-county records of all regions (Table 1, Figure 1). Tukey's means separation test was used to compare regions when significant in an ANOVA (*P* < 0.05, 48). The agro-landscape and weather conditions were compared across regions with the same procedure. Data transformations appropriate for count data with zeroes (square root of value + 0.5) and proportion data (arcsine of the square root of value) were done before analyses. Transformations selected were standard to compensate for variation from the ANOVA normality assumption associated with these measurements (48). Untransformed means were presented.

Correlation analyses for data records in each region were conducted [Proc Corr, (49)]. Using transformed data to address possible deviations from normality, Pearson correlations of natural enemy-*M. sorghi* abundance were used to assess numerical response of selected natural enemy taxa to *M. sorghi* abundance. The natural enemy activity correlations with agro-landscape and weather metrics were used to explore potential mediation of natural enemy activity by landscape and weather conditions. Five probability levels (*P* < 0.20, *P* < 0.10, *P* < 0.05, *P* < 0.01, *P* < 0.001) were used for detecting patterns and contrasts of correlations across regions. The Holm-Bonferroni method (50) was used to sequentially adjust each calculated probability value to compare to the five set critical probability levels for the correlations across regions where each natural enemy taxon occurred (i.e., four correlations of four regions, except the case of *L. testaceipes* that was not detected in one region). Of interest were the natural enemy abundance-*M. sorghi* abundance correlations, natural enemy activity-agro-landscape correlations, and natural enemy activity-weather correlations. In lieu of structured experiments to test for potential interactions and collinearity of the agro-landscape and weather variables, the five probability levels allowed for flexibility in considering variables for further multivariate regression model activities (48) for regions of special interest based on the outcomes of regional differences and risk scenario comparison of this study.

## Results

### *Melanaphis sorghi* and Natural Enemy Taxa Across Regions

*Melanaphis sorghi* was detected in all regions and years. Presence of the family-level taxa and species of natural enemies were nearly identical across the four regions (Table 2). Two primary endoparasitoids and four of six coccinellid species were common. Syrphidae, Chrysopidae, and Hemerobiidae were detected in all regions, with eight species common in the South and South GP regions where species were identified (Table 2). These taxa were detected each year. In contrast to the nearly identical natural enemy complex across regions, selected insect abundance and activity varied across years and regions. Yearly variation in abundance of *A. nigritus* mummies and larvae of syrphids and lacewings was detected (the ANOVAs for the three taxa were significant, for the three *F* > 5.3; d.f. = 4, 70; *P* < 0.001), while other abundance metrics and all natural enemy activity metrics adjusting for *M. sorghi* density did not experience significant year-to-year variation (*P* > 0.05) (description of year, region, and field identifiers are given in Supplementary Material 1). Although yearly variation was modest, the year term was retained in the ANOVAs to aid partitioning year-to-year variation from the error term used to test for regional differences and to maintain a common analysis structure for all measurements.

**Table 2:** Natural enemy taxa present in four regions where *Melanaphis sorghi* and its natural enemies were identified to family and species when possible, from 2015 to 2019.

**Family^a^**	**Species^a^**	**South^b^**	**South GP^b^**	**South E^b^**	**North GP^b^**
Aphelinidae		X	X	X	X
	*Aphelinus nigritus* Howard	X	X	X	X
Braconidae		X	X	X	X
	*Lysiphlebus testaceipes* (Cresson)	X	X	X	–
Coccinellidae		X	X	X	X
	*Coccinella septempunctata* L.	X	X	X	X
	*Coleomegilla maculata* (De Geer)	X	X	X	X
	*Cycloneda sanguinea* (L.)	X	X	X	–
	*Harmonia axyridis* (Pallis)	X	X	X	X
	*Hippodamia convergens* Guérin-Méneville	X	X	X	X
	*Olla v*-*nigrum* (Mulsant)	X	X	–	–
	Scymninae	X	X	X	nd
Syrphidae		X	X	X	X
	*Allograpta obliqua* (Say)	X	X	X	nd
	*Eupeodes americanus* (Wiedemann)	X	X	–	nd
	*Dioprosopa clavata* (F.)	X	X	X	nd
Chrysopidae		X	X	X	X
	*Ceraeochrysa valida* (Banks)	X	X	X	nd
	*Chrysopa quadripunctata* Burmeister	X	X	nd	nd
	*Chrysoperla externa* (Hagen)	X	X	nd	nd
	*Chrysoperla rufilabris* (Burmeister)	X	X	nd	nd
	*Chrysoperla plorabunda* (Fitch)	X	X	nd	nd
Hemerobiidae		X	X	X	X

*In columns, ‘X’ indicates detected, ‘—’ indicates not detected, ‘nd’ indicates family detected but species determination not made.*

a *Order-level taxa: Hymenoptera: Aphelinidae and Braconidae; Coleoptera, Coccinellidae; Diptera, Syrphidae; Neuroptera, Chrysopidae and Hemerobiidae).*

b *South: Texas coastal areas from Rio Grande Valley to near Houston; South GP, central Texas to lower Texas Panhandle and across central Oklahoma; North GP, upper Texas and Oklahoma Panhandles through northern Oklahoma and adjacent southern Kansas; South E, southern and central Alabama*.

**Figure 2:**
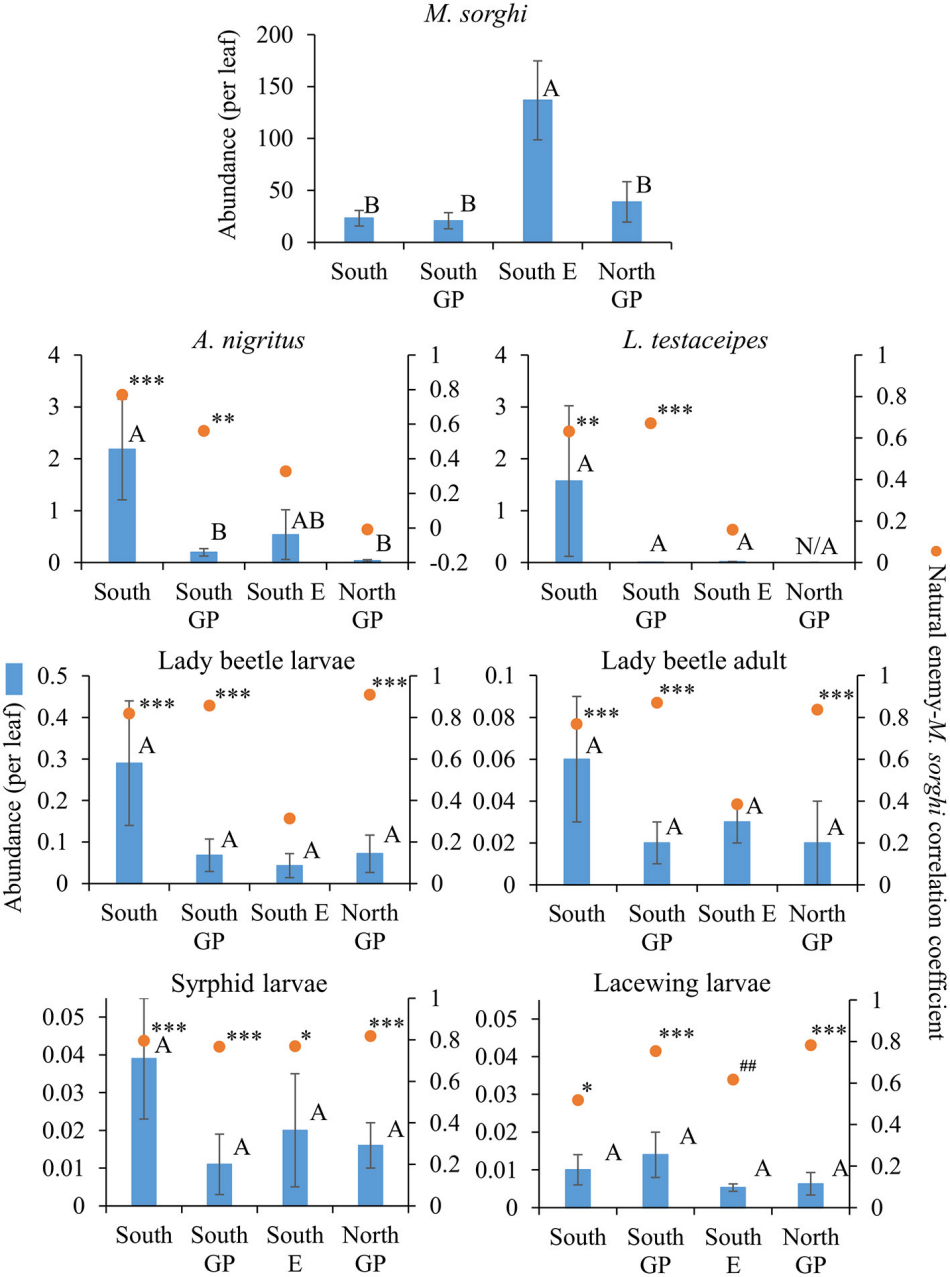
Average (±SEM) abundance (per leaf) (blue bars) of *Melanaphis sorghi* and natural enemies in each of the four regions from 2015 to 2019. Lack of significant differences across regions indicated by common letters (α = 0.05, Tukey's mean separation test, each taxon analyzed separately). Dots represent natural enemy abundance-*M. sorghi* abundance correlations, using five probability levels (‘#’ = *P* < 0.20, ‘##’ = *P* < 0.10, ‘*’ = *P* < 0.05, ‘**’ = *P* < 0.01, ‘***’ = *P* < 0.001) for pattern detection across regions. The Holm-Bonferroni method (50) was used to adjust the calculated probabilities to compare to these levels across the four regions. See Figure 1 for geographical boundaries of the regions.

Measuring abundance, significant differences across regions were detected for *M. sorghi* (*F* = 7.08; d.f. = 3, 79; *P* = 0.0003) and *A. nigritus* (*F* = 6.28; d.f. = 3, 79; *P* = 0.0007) but differences were not detected for other taxa (*P* > 0.05) (Figure 2). Abundances of *A. nigritus, L. testaceipes*, lady beetles, and syrphids were two- to over 10-fold greater in the South region than other regions, although variation was high and only *A. nigritus* abundance in the South was significantly greater than in other regions (Figure 2). *Melanaphis sorghi* was most abundant in the South E region where natural enemy abundance, except *A. nigritus*, was relatively low (Figure 2).

**Figure 3:**
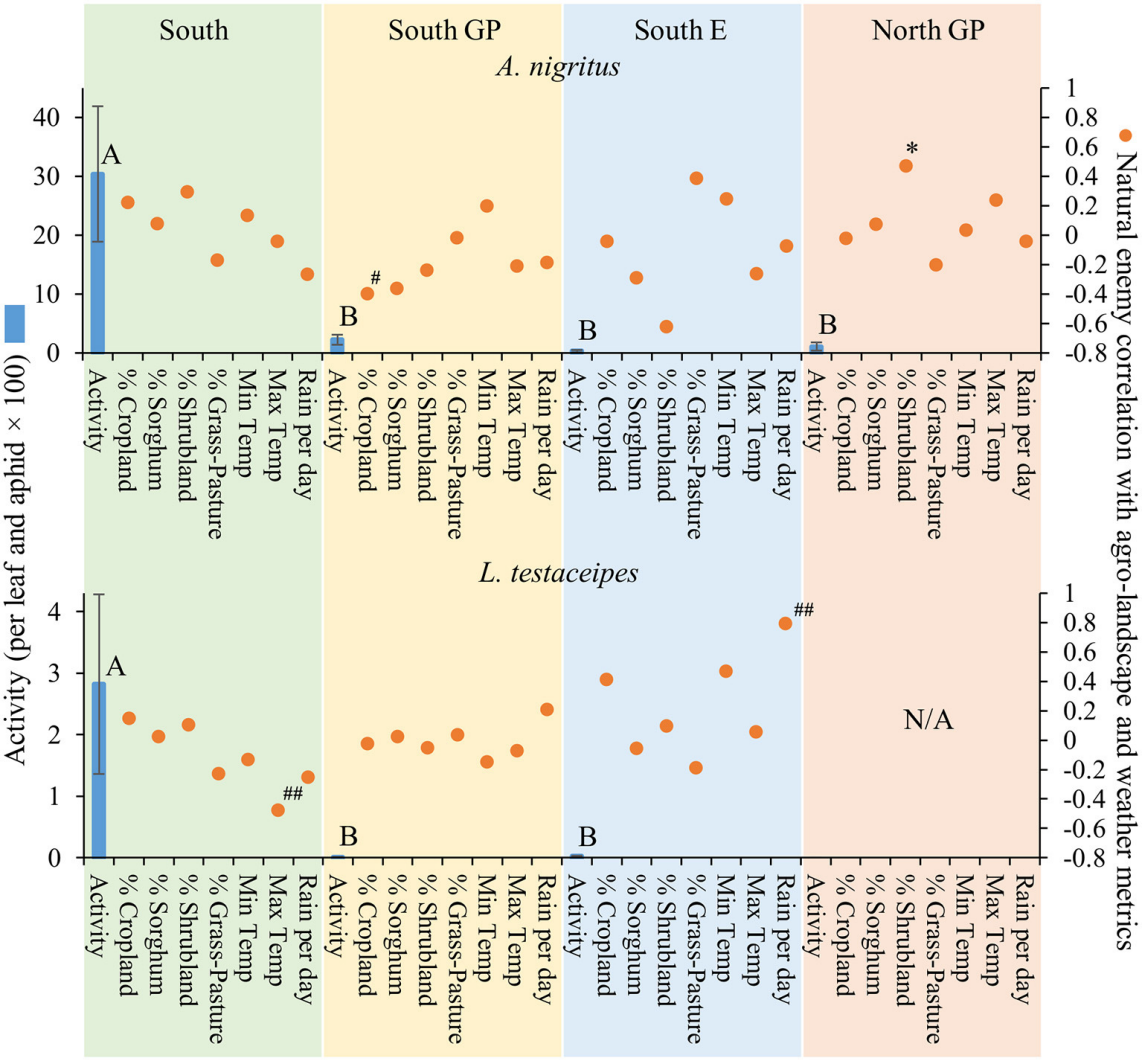
Average (±SEM) activity of *Aphelinus nigritus* and *Lysiphlebus testaceipes* (per leaf and aphid x 100) (blue bars) in each of the four regions from 2015 to 2019. Lack of significant differences across regions indicated by common letters (α = 0.05, Tukey's mean separation test, each taxon analyzed separately). Dots represent natural enemy activity-agro-landscape correlations, and natural enemy activity-weather correlations, using five probability levels (‘#’ = *P* < 0.20, ‘##’ = *P* < 0.10, ‘*’ = *P* < 0.05, ‘**’ = *P* < 0.01, ‘***’ = *P* < 0.001) for pattern detection across regions. The Holm-Bonferroni method (50) was used to adjust the calculated probabilities to compare to these levels across the regions. See Figure 1 for geographical boundaries of the four regions.

**Figure 4:**
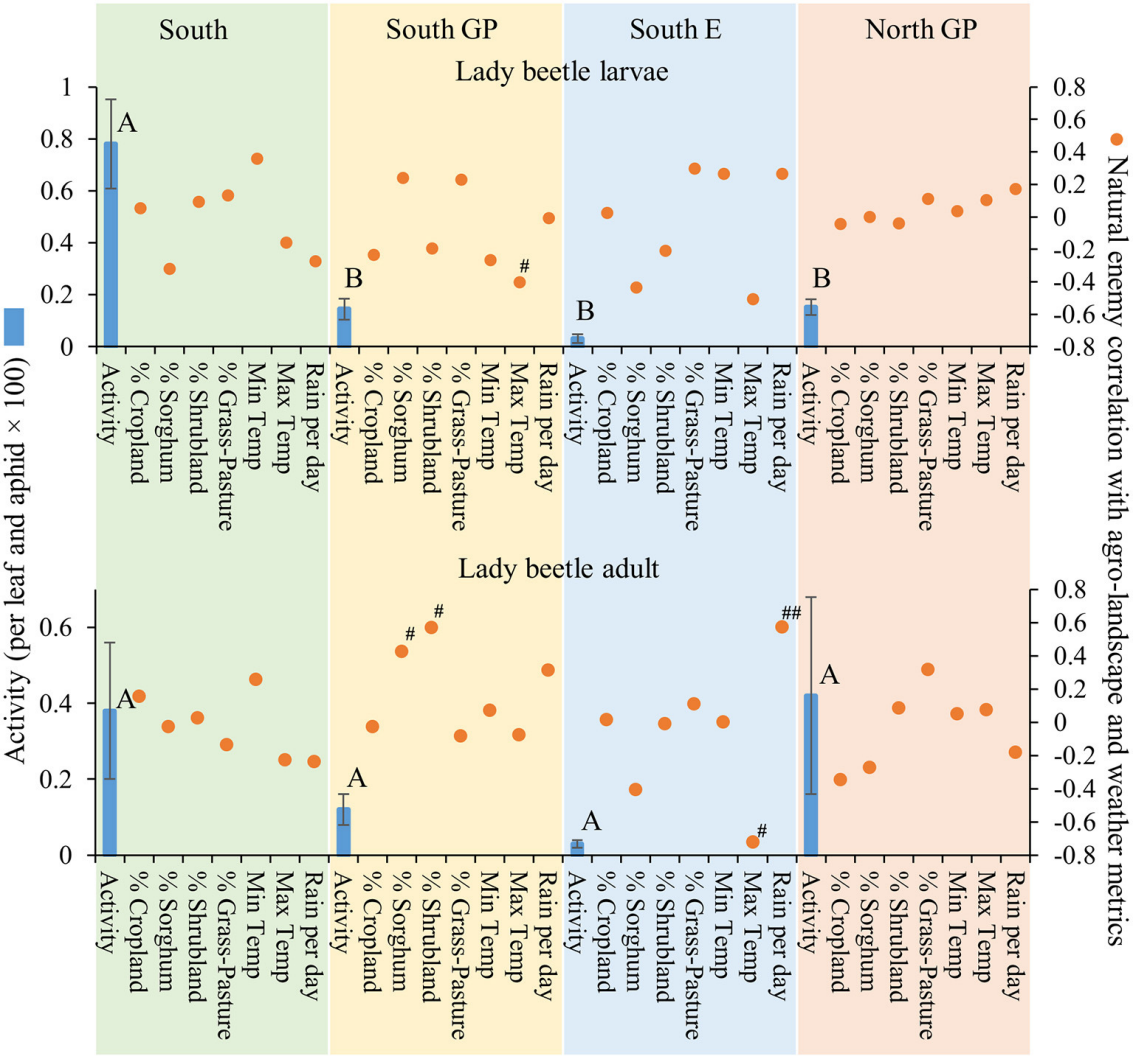
Average (±SEM) activity of lady beetle larvae and adults (per leaf and aphid x 100) (blue bars) in each of the four regions from 2015 to 2019. Lack of significant differences across regions indicated by common letters (α = 0.05, Tukey's mean separation test, each taxon analyzed separately). Dots represent natural enemy activity-agro-landscape correlations, and natural enemy activity-weather correlations, using five probability levels (‘#’ = *P* < 0.20, ‘##’ = *P* < 0.10, ‘*’ = *P* < 0.05, ‘**’ = *P* < 0.01, ‘***’ = *P* < 0.001) for pattern detection across regions. The Holm-Bonferroni method (50) was used to adjust the calculated probabilities to compare to these levels across the regions. See Figure 1 for geographical boundaries of the four regions.

**Figure 5:**
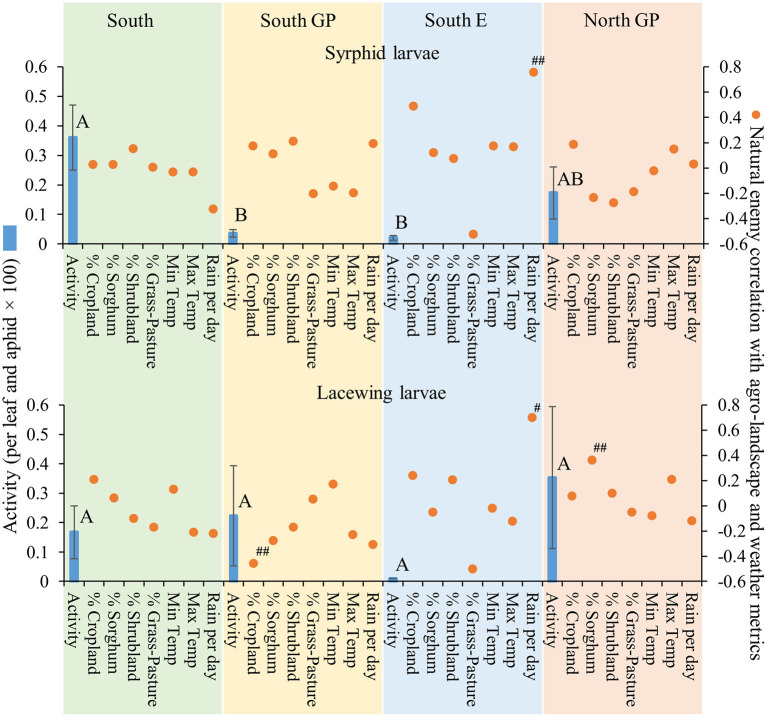
Average (±SEM) activity of syrphid and lacewing larvae (per leaf and aphid x 100) (blue bars) in each of the four regions from 2015 to 2019. Lack of significant differences across regions indicated by common letters (α = 0.05, Tukey's mean separation test, each taxon analyzed separately). Dots represent natural enemy activity-agro-landscape correlations, and natural enemy activity-weather correlations, using five probability levels (‘#’ = *P* < 0.20, ‘##’ = *P* < 0.10, ‘*’ = *P* < 0.05, ‘**’ = *P* < 0.01, ‘***’ = *P* < 0.001) for pattern detection across regions. The Holm-Bonferroni method (50) was used to adjust the calculated probabilities to compare to these levels across the regions. See Figure 1 for geographical boundaries of the four regions.

Natural enemy activity (adjusted for *M. sorghi* abundance) supported that the parasitoids, and selected predators to a lesser extent, responded numerically to *M. sorghi* differently across regions. Specifically, the activity metric of the two parasitoids and larvae of lady beetles and syrphids varied across regions (the ANOVAs for the four taxa were significant, for the four *F* > 2.8; d.f. = 3, 79; *P* < 0.05). The activity metrics of the two parasitoids (*A. nigritus, L. testaceipes*) (Figure 3) and lady beetle larvae (Figure 4) were greatest in the South region, and syrphid activity was relatively high in the South region but did not differ significantly from the North GP region (Figure 5). Related, high field parasitism rates (35 to 90%) attributed to *A. nigritus* and *L. testaceipes* were periodically observed in the South region (MJB and AMF, pers. obs.). High variance relative to the mean was observed for *L. testaceipes* in the South region (Figure 2), where occasional *L. testaceipes* parasitism exceeding 90% was observed in 2017 onward (MJB and AMF, pers. obs.).

### Enemy-*M. sorghi* Associations Mediated by Agro-Landscape and Weather Conditions

Relevant to interpreting the correlation analyses, significant differences were detected across regions for the agro-landscape and weather metrics (the ANOVAs for the seven metrics were significant, for the seven *F* > 3.70; d.f. = 3, 79; *P* < 0.02) (Table 3). Year-to-year variation in minimum temperature was detected (*F* = 4.82; d.f. = 4, 70; *P* = 0.0017), while no significant year-to-year variation in other agro-landscape and weather metrics was detected (*P* > 0.05).

**Table 3:** Average agro-landscape features and weather conditions experienced by *Melanaphis sorghi* and natural enemies across four regions, from 2015 to 2019.

		**Agro-Landscape Feature^b^**				**Weather^c^**		
**Region^a^**	***N***	**% Crop**	**% Sorghum**	**% Shrub**	**% Grass-Pasture**	**Min Temp**	**Max Temp**	**Rainfall (x100)**
South	21	72.7 ± 6.1a	29.8 ± 4.1a	3.70 ± 0.68ab	7.78 ± 2.10b	24.2 ± 0.52a	33.3 ± 0.36ab	2.03 ± 0.86a
South GP	27	51.3 ± 4.3b	2.72 ± 0.59bc	4.71 ± 1.49b	28.6 ± 3.13a	20.8 ± 0.43b	34.1 ± 0.40a	3.15 ± 0.54a
South E	9	27.4 ± 5.9b	0.13 ± 0.06c	10.3 ± 1.41b	5.66 ± 0.92b	20.1 ± 1.1bc	32.1 ± 0.46b	3.38 ± 1.33a
North GP	30	47.8 ± 3.8b	4.59 ± 0.89b	2.79 ± 1.06a	37.0 ± 3.53a	18.8 ± 0.38c	32.5 ± 0.28b	7.60 ± 0.77a

*Lack of significant differences across regions (down columns) indicated by common letters (α=0.05, Tukey's mean separation test).*

a *South, Texas coastal areas from Rio Grande Valley to near Houston; South GP, central Texas to lower Texas Panhandle and across central Oklahoma; North GP, upper Texas and Oklahoma Panhandles through northern Oklahoma and adjacent southern Kansas; South E, southern and central Alabama.*

b *Landscape metrics extracted from cropland data layers by year and location [Cropscape, (41)]. Metrics calculated from a buffer of 5 km with sampling location used as the centroid. See text for definitions of the agro-landscape metrics.*

c *Weather conditions taken from the nearest weather station to each sampling event (47). Minimum (Min) and maximum (max) temperature (°C) is average of daily lowest and highest temperatures over the course of sampling. Rain per day is the average rainfall (cm) per day over the course of sampling*.

As indicated by percent composition of the 5 km buffers of the sampling locations, sorghum and cropland in general were more concentrated in the South region, followed by the two Great Plains regions. Cropland was a substantial landscape component in the South E region, but sorghum was less represented (Table 3 and Figure 1). In contrast, shrubland was common in the South E region, and percent grassland-pasture was about four- to five-fold higher in the South GP and North GP regions than the other two regions. These metrics generally aligned with the historical cropping patterns of the areas sampled (1). Differences were detected for weather metrics across the regions and generally followed a latitudinal gradient of warmer minimum and greater maximum daily temperatures of the subtropical-influenced regions (South and South E regions) compared with the two temperate-influenced Great Plains regions (South GP and North GP regions) (Table 3). The South GP region reached relatively high maximum temperatures as likely influenced by inland weather patterns where sampling occurred. Average rainfall across sampling events did not differ across the regions, although variable rainfall occurred in the South E region as reflected in the high variance relative to the mean (about 40% of the mean) (Table 3).

Different correlation patterns of natural enemy abundance with *M. sorghi* abundance, and natural enemy activity with agro-landscape and weather conditions, were observed across regions. The South region experienced low average *M. sorghi* densities (~23 *M. sorghi* per leaf) (Figure 2) where activities of both parasitoids and lady beetle larvae were high (Figures 3, 4). All natural enemy-*M. sorghi* abundance correlations were significant and positive using the Holm-Bonferroni adjustment of probability values (Figure 2). In this region, *M. sorghi*-natural enemy interactions appeared to be functioning independent of or sufficiently robust to mask any agro-landscape and weather influences, as indicated by the lack of significant correlations of natural enemy activity with these metrics (Figures 3–5). In the South GP region, *M. sorghi* abundance was also low (~21 *M. sorghi* per leaf), and all natural enemy-*M. sorghi* abundance correlations were significant and positive in sign (Figure 2). The potential influence of agro-landscape composition on parasitism and predation of selected taxa was more apparent in this region: *A. nigritus* (Figure 3) and lacewing larvae (Figure 5) were negatively correlated with percent cropland, while lady beetle adults (Figure 4) were negatively correlated with percent shrubland and sorghum.

In contrast, *M. sorghi* abundance approached or exceeded the 40 *M. sorghi* per leaf indicator of economic concern [i.e., the economic threshold (18)] in the North GP and South E regions (~38 and ~130 *M. sorghi* per leaf, respectively) (Figure 2). In the North GP region, significant natural enemy-*M. sorghi* abundance correlations were limited to predators (Figure 2). In this region, natural enemy activity mediated by agro-landscape conditions was detected: one significant positive correlation of *A. nigritus* activity with percent shrubland (Figure 3) and another of lacewing larval activity with percent sorghum (Figure 5). *Lysiphlebus testaceipes* was not observed parasitizing *M. sorghi* in the North GP region (Figure 2). In the South E region, natural enemy abundance and activity were generally low (Figure 2 through Figure 5). Significant correlations of natural enemy-*M. sorghi* abundance were limited to syrphid and lacewing larvae (Figure 2). In this region, natural enemy activity was mediated in part by weather. Increasing rainfall was associated with greater *L. testaceipes* parasitism (Figure 3), lady beetle adults (Figure 4), and syrphid and lacewing activity (Figure 5). Increasing maximum temperature was associated with decreasing lady beetle adult activity (Figure 4). In contrast, only two significant natural enemy-weather correlations of a total of 51 were detected in the other regions (Figure 2 through Figure 5).

## Discussion

### Comparison of Natural Enemy Data to Previous Observations

The parasitoid and predator species complex in the South region and two Great Plains regions did not change from those previously reported in central and south Texas (23, 26) and overlapped and added to those observed in Kansas (22). A few additional braconid parasitoid species, such as *Aphidius* spp., and several additional species of coccinellids and syrphids were reported farther south in northeastern to westcentral Mexico (24, 25, 39, 51). In the South E region, family-level taxa of predators reported in this study were consistent with those previously reported (38, 52). Overall, the natural enemy complex was composed of a broad array of taxa observed across 5 years and four regions that were present over much of the geographic range of *M. sorghi* on sorghum in North America.

Regional differences in natural enemy abundance and activity helped place in perspective earlier reporting of parasitism and predation of *M. sorghi*. The high *A. nigritus* activity metric of the South region (Figure 3) equated to a study-long average of ca. 23% apparent percent parasitism and was coupled with low *M. sorghi* abundance (Figure 2). This activity was comparable to 31% parasitism of *M. sorghi* attributed to several species in central Mexico (24). Variable parasitoid abundance and activity (Figures 2, 3) were consistent with episodes of high *A. nigritus* and *L. testaceipes* parasitism in the South region (MJB and AMF, pers. obs.). Colares et al. (22) in Kansas also detected *M. sorghi* suppression by *Aphelinus* spp. using *M. sorghi*-infested potted sorghum. They did not observe *L. testaceipes* parasitism of *M. sorghi*, which corresponded to lack of *L. testaceipes* parasitism of *M. sorghi* observed in the North GP region (Figure 2). It is notable that *L. testaceipes* was observed parasitizing *S. graminum* and *R. maidis* that occurred sporadically in the North GP region (NCE, pers. obs.), but *M. sorghi* tan mummies likely indicative of the braconid *L. testaceipes* were not detected (Figure 2). The very low parasitoid and predator activities in the South E region across a wide range of aphid densities were consistent with little to no parasitism previously observed in Georgia and Kentucky (38, 52). These findings supported the interpretation that natural enemies were contributing to *M. sorghi* suppression in the South region, to a lesser extent in the two Great Plains regions, and not in the South E region.

### Regional Comparisons and Sorghum Risk Scenarios

Soon after the *M. sorghi* invasion, ecological modeling and qualitative evaluations of sorghum risk initially focused on aphid life history, extent of sorghum cultivation and susceptibility to *M. sorghi*, and weather (APW risk assessment scenario). This scenario was useful in evaluating geographic and annual range expansion of *M. sorghi* (19, 20). The more comprehensive AE/LW risk scenario proposed here drew from the enemy-free space hypothesis (13) and the allied biotic interference hypothesis in the biological control literature (53) to explain *M. sorghi* outbreaks when natural enemies were lacking, as well as reduced risk of outbreaks when natural enemies numerically responded to *M. sorghi* (54). The AE/LW risk scenario also incorporated potential influence of weather on *M. sorghi*-natural enemy interactions based in part on climate suitability (14) among the different regions. Last, bottom-up forces (11, 12) were considered within the context of agro-landscape composition elements (i.e., sorghum, crops collectively, and less managed grassland/shrubland) including serving as harborage for natural enemies of cereal aphids in the North American Great Plains and elsewhere (6, 29, 32, 33, 55, 56).

Applied to the current study, the AE/LW risk scenario accounted for natural enemy abundance and activity that was highest in the South region, functioned well across agro-landscape and weather conditions, and was accompanied by low *M. sorghi* abundance (~23 *M. sorghi* per leaf) (Figure 2 through Figure 5). These findings and observations that the South region experienced few *M. sorghi* outbreaks during the study (MJB, pers. obs.) were inconsistent with the APW risk scenario that predicted high sorghum risk (Figure 1). Positive correlations of natural enemy-*M. sorghi* abundance were detected for each natural enemy taxon in the South and South GP regions where *M. sorghi* abundance was low (~20 *M. sorghi* per leaf) (Figure 2). Also consistent with the AE/LW risk scenario, natural enemy activity of selected taxa appeared to be mediated by landscape composition particularly in the South GP region where *M. sorghi* abundance was low, and by weather particularly in the South E region where *M. sorghi* abundance was highest of the regions (~136 aphids/leaf) (Table 3 and Figure 3 through Figure 5). Although risk expectations of the two scenarios were similar in the two Great Plains regions and the South E region, potential mediation of *M. sorghi*-natural enemy interactions by landscape composition and weather conditions was revealed by considering the AE/LW risk scenario. Broadly, the AE/LW risk scenario appeared suited, and essential in the South region, in assessing risk of pest outbreaks on a regional scale. These findings set the stage for producing risk models with agroecosystem response estimates under current (i.e., extant regions of this study) and changing conditions (i.e., anticipated under climate change) considered in the AE/LW risk scenario.

Prior knowledge of natural enemies of cereal aphids and agro-landscape influences also supported the idea that natural enemies as top-down forces in *M. sorghi* regulation contribute to shaping sorghum agroecosystem response to the *M. sorghi* invasion, as seen in past cereal aphid invasions in the North American Great Plains (6). On wheat, parasitism by *L. testaceipes* was sufficient to prevent populations of *S. graminum* from reaching damaging population levels (57), and parasitism was positively correlated with plant diversity (cropland and grassland/pasture) (32). Parasitism of *D. noxia* by *Aphelinus albipodus* Hayat and Fatima (Hymenoptera: Aphelinidae) was associated with declines in aphid abundance, especially where wheat production was nested in a relatively diverse cropping system (30) and planted with sunflower in a strip cropping system (55). Outside of the Great Plains, Thies et al. (33) studied parasitoids of cereal aphids affected by landscape diversity in Europe and concluded that increasing landscape diversity was associated with both increasing parasitoid abundance and increasing cereal aphid abundance.

From a perspective of managing pests in individual fields, parasitism (*L. testaceipes* mummy detection) has been incorporated into *S. graminum* monitoring and management protocols in Oklahoma (57). From a regional or areawide pest management perspective, this study adds to the evidence that parasitism and predation by resident natural enemies are well suited to aphid management in the low-input large-scale cereal agroecosystem of the North American Great Plains (6, 29, 30). For *M. sorghi*, combining an agroecosystem service of parasitism and predation with use of sorghum resistant to *M. sorghi* that does not deter parasitism and predation (23, 26) is particularly important where risk is more persistent, variable, or prone to variable natural enemy activity as mediated by agro-landscape and weather conditions (e.g., South E and South GP regions). In such cases, variable top-down forces provided by natural enemies benefit from the addition of bottom-up forces of sorghum resistant to *M. sorghi*, as well as strategic use of insecticides with low toxicity to natural enemies (26, 58).

### Considerations for Agroecosystem Response Modeling

This region-scale pest risk assessment approach of gathering insect sampling data for considering differences in *M. sorghi* outbreak risk warrants further effort, such as how to best utilize large region trends for addressing risk assessment needs at smaller spatial scales. To this end, correlation patterns identified of potential interest in the large region comparisons may be explored further by considering new or component data used to derive the metrics of this study. For example, agro-landscape metrics not considered in the current study, such as configuration of landscape elements (e.g., edge density and plant diversity metrics) and scale-suitability of the array of natural enemies, may help resolve the correlation patterns of mixed sign that were occasionally detected (i.e., natural enemy activity correlated with landscape composition metrics as seen in the south GP region, Figure 3 through Figure 5) (59). These metrics may be further explored in a GIS using the agro-landscape data layers of the current study (Figure 1). Further multivariate regression modeling, such as stepwise regression or regression modeling with collinearity testing, may generate estimates of the degree of influence of natural enemies under varying agro-landscape and weather conditions considered in the AE/LW risk scenario. We note that collinearity among variables may affect sensitivity of model distinction between regions because of its influence on coefficients and model probability. But for models selected within any region of special interest, multicollinearity is less relevant to mean responses of the dependent variable and prediction (i.e., aphid and natural enemy abundance, and natural enemy activity) (48). The variable selection process of stepwise regression provides additional safeguards (48). Should large sets of landscape and weather metrics be added, such as landscape configuration and diversity metrics, other multivariate techniques such as principal component analysis may be advised to obtain a synthetic smaller set of variables that retains the essential information of the large data set (60).

Additionally, Chaplin-Kramer et al. (56) noted the value and under-use of time sequences of pest and natural enemy monitoring data to make inferences on suppression by comparing population growth rates of the pest and natural enemies. Our use of the current data set averaged this time sequence feature (Table 1) which facilitated the four region comparison and the contrast of the two risk scenarios. For an analytical focus on suppression by natural enemies over time, the component data from all fields and sampling events are available (Supplementary Material 1). Such analyses should consider the possibility of autocorrelation of data across the time sequences and range of the time sequences in the data set. Last, the region-scale risk assessment approach of this study can help guide and prioritize companion structured experimentation. For example, natural enemy exclusion experiments conducted in the South region estimated up to 90% suppression of *M. sorghi* by *A. nigritus* and coccinellids (61).

### Perspectives in Regional Risk Assessment and Agroecosystem Response to Invaders

Previous ecological modeling and qualitative sorghum risk evaluations focused on the APW risk scenario for *M. sorghi* and did not include top-down constraints provided by natural enemies. The focus on climate suitability (14), resource availability, and defense-free space afforded by growing sorghums with high yield potential but susceptible to *M. sorghi* [i.e., widespread cultivation of sorghums susceptible to *M. sorghi* (11, 12)] and mechanisms of rapid range expansion (19, 20) had value in evaluating geographic and annual range expansion of *M. sorghi* and the overall initial success of the *M. sorghi* invasion in North America. Regarding the allied interests of invasive species ecology and biological control (53), the AE/LW risk scenario proposed here added the top-down force of natural enemies as a key element of biotic resistance to *M. sorghi* (13, 17) to explain the region-specific risk of *M. sorghi* in the context of potential influence of agro-landscape and weather conditions across a near-continent scale of the *M. sorghi* invasion.

These findings have implications for climate change affecting plant-aphid-natural enemy interactions that result in change to agroecosystem-based pest management. Triltsch et al. (62) projected that a temperature increase of 3°C would result in reduction of cereal aphid infestations of wheat due to quicker wheat seed maturation, increased predator rates, and lower aphid reproduction rate. Alternatively, parasitism by some species may be adversely affected or remain unchanged while an increase in the host aphid's reproductive rate increases, indicating the species dependency of such predictions (63). The presence of *M. sorghi* across subtropical and temperate latitudes in North America allows for proposing risk hypotheses as temperate zones of this study warm. Higher *M. sorghi* abundance may be expected under the APW risk scenario based on warming trend predictions of climate change in the more northern temperate zone range of *M. sorghi*. But the potential of natural enemy influences of warming temperature in the context of landscape structure provided under the AE/LW sorghum risk scenario should be considered given species dependent effects of warming temperatures (63). Given the similar natural enemy complex across the latitudinal gradient of this study, sorghum-*M. sorghi*-natural enemy dynamics of the South GP and potentially North GP regions may shift toward lower *M. sorghi* abundance and higher natural enemy activity as seen in the subtropical influenced South region (Figure 2). One consideration is lag time of climate change effects on landscape structure, particularly managed lands, although such change may have long-term influence.

The AE/LW risk scenario appears more suitable now that the expansion area of *M. sorghi* appears stable (6). At least in the South region of this study and farther south into Mexico (38), the AE/LW risk scenario appears essential to account for low *M. sorghi* abundance and high natural enemy activity, where higher *M. sorghi* abundance and sorghum risk were expected under the APW risk scenario given its focus on climate suitability of sorghum and *M. sorghi*. Large region trends from the AE/LW risk scenarios may be used for general guidance in recommending supportive management inputs to complement the agroecosystem service of parasitism and predation (Figure 1). Further refinement and quantification of outcomes of the AE/LW sorghum risk scenario may be aided by deep analysis of the decomposed year-county data records, and further modeling of agroecosystem response under current and changing conditions considered in the AE/LW risk scenario. These data may also contribute to meta-analysis efforts that focus on agroecosystem resilience to invasive species [e.g., (56, 64)] and understanding biotic resistance to insect invasions within the context of general influence of abiotic factors (12, 13, 17) that vary across large-scale agroecosystems. Based on this case study, the AE/LW risk scenario appears suited and flexible in assessing risk of pest outbreaks regionally and improves understanding agroecosystem response to invasive insect species where natural enemies vary in their abundance and interaction with *M. sorghi* along gradients of agro-landscape and weather conditions.

## Data Availability Statement

The original contributions presented in the study are included in the article Supplementary Material, further inquiries can be directed to the corresponding authors.

## Author Contributions

MB and NE contributed to conception and design of the study. MB, NE, IE, AJ, AF, and BE organized the database. MB performed the statistical analysis and wrote the first draft of the manuscript. All authors contributed to manuscript revision, read, and approved the submitted version.

## Funding

We thank the United Sorghum Checkoff Program for financial support. The USDA Agricultural Research Service also provided funding through the Areawide Pest Management Program, project 3072-22000 (017-03-S, 017-05-S, and 017-04-S). Funders evaluated and provided comments on application for funding. This research was conducted partly under the umbrella of USDA NIFA Hatch project TEX0-2-9394 assigned to MB at Texas A&M AgriLife Research.

## Conflict of Interest

JG was employed by Syngenta Crop Protection. The remaining authors declare that the research was conducted in the absence of any commercial or financial relationships that could be construed as a potential conflict of interest.

## Publisher's Note

All claims expressed in this article are solely those of the authors and do not necessarily represent those of their affiliated organizations, or those of the publisher, the editors and the reviewers. Any product that may be evaluated in this article, or claim that may be made by its manufacturer, is not guaranteed or endorsed by the publisher.
